# Effects of dietary supplementation of golden apple snail (*Pomacea canaliculata*) egg on survival, pigmentation and antioxidant activity of Blood parrot

**DOI:** 10.1186/s40064-016-3051-2

**Published:** 2016-09-13

**Authors:** Song Yang, Qiao Liu, Yue Wang, Liu-lan Zhao, Yan Wang, Shi-yong Yang, Zong-jun Du, Jia-en Zhang

**Affiliations:** 1 College of Animal Science and Technology, Sichuan Agricultural University, Chengdu, 611130 Sichuan China; 2 Institute of Tropical and Subtropical Ecology, South China Agricultural University, Guangzhou, 510642 Guangdong China

**Keywords:** Golden apple snail egg, Carotenoid, Blood parrot, Pigmentation, Antioxidant activity

## Abstract

This study aims to evaluate the effects of supplementing golden apple snail (*Pomacea canaliculata*) eggs powder (EP) in the diet as a source of natural carotenoids on survival, pigmentation and antioxidant activity of Blood parrot. A total of 90 fish were divided into three treatment groups with three replicates per treatment. Blood parrot were fed with diets containing 0 (control), 5 % (EP 5 %), and 15 % (EP 15 %) dry powder of golden apple snail egg for 60 days, and nine fish per group were sampled at 20, 40, and 60 days. No differences in survival of the fish among treatments were found throughout the experiment. The body coloration of Blood parrot was enhanced in the skin and caudal fin with increasing content of golden apple snail egg powder in the diet. At the end of the experiment, the carotenoid content in the caudal fin and the number of scale chromatophores of the fish fed dietary with EP were higher (*P* < 0.05) than those of the control group. The EP 15 % treated fishes showed a significant higher (*P* < 0.05) in the activities of SOD after 60 days, but we could not observe significant changes (*P* > 0.05) in CAT activities. Results demonstrated that golden apple snail eggs can be used as a colorant to promote the pigmentation efficacy of Blood parrot.

## Background

Blood parrot is a hybrid of female *Cichlasoma synspilum* and male *Cichlasoma citrinellum* artificially cross-bred in Taiwan as early as 1980s (Yang et al. 2012). Currently, Blood parrot is one of the most popular and high-value ornamental fish because of its bright body color, their size is often 10–12 cm. At present, they can be breed and produced with commercial feed in fish farm. Nonetheless, the fish cannot synthesize their own carotenoid color. As the body color is the major factor that affects the market value of ornamental fish, enhancing it by supplementing pigments in diets is therefore necessary (Gouveia et al. 2003; Mills and Patterson 2009; Olson and Owens 1998). Astaxanthin is a pigment widely used in the foodstuff and forage industries as a dietary supplement for developing prospects (Spiller and Dewell 2003). In aquaculture, the carotenoids can be produced synthetically and are commonly used for pigmentation of fishes; furthermore, alternative natural carotenoid sources (yeast, algae, higher plants, and crustacean meal) have also been studied (Büyükçapar et al. 2007; Chatzifotis et al. 2011; Teimouri et al. 2013; Kalinowski et al. 2007; Lee et al. 2010; Pham et al. 2014; Wang et al. 2006; Whyte and Sherry 2001). In addition, carotenoids were reported to be able to change antioxidant activity of fish (Pham et al. 2014; Wang et al. 2006).

Golden apple snail (*Pomacea canaliculata*) is a freshwater gastropod that has become a serious pest of agriculture and included in the world’s 100 worst invasive alien species (Lowe et al. 2000). This species has invaded several European, North American, and Asian countries and damages rice and aquatic organisms (Accorsi et al. 2014; Horgan et al. 2014; Karraker and Dudgeon 2014). Given the adverse effect of this species, physical, chemical, and biological control techniques have been established; such methods include crop rotation (Wada et al. 2004), use of molluscicides (Cruz et al. 2000; Quijano et al. 2014), and use of predators (Su Sin 2006; Ip et al. 2014; Yusa et al. 2006). As an abundant aquatic living resource, utilization of egg of golden apple snails has been rarely reported.

This snail eggs cemented outside water present bright colors and contain 72 nmol carotenoids per gram (Dreon et al. 2004). The major carotenoid was astaxanthin in its free (40 %), monoester (24 %), and diester (35 %) forms, mainly esterified with 16:0 fatty acid (Dreon et al. 2004). Previous studies reported that natural or synthetic astaxanthin can be used as an additive to enhance fish body coloration and antioxidant activity (Ho et al. 2013; Yang et al. 2012; Yedier et al. 2014). Furthermore, additions dietary of astaxanthin extracted from golden apple snail eggs showed efficiency for improved skin pigmentation in fancy carp (*Cyprinus carpio*) (Boonyapakdee et al. 2014). Despite its suitability as an additive, the high production cost of astaxanthin limits its commercial application on the large scale (Yang et al. 2012). Thus, the use of golden apple snail eggs would benefit to aquaculture and agriculture industry.

This study was conducted to evaluate the effects of adding golden apple snail eggs as a dietary carotenoid source on the survival, pigmentation and antioxidant activity of Blood parrot.

## Methods

All experimental procedures and sample collection were approved by the Institutional Animal Care and Use Committee (IACUC) of the College of Animal Science and Technology of Sichuan Agricultural University, Sichuan China, under permit No. DKY-B20121403.

### Experimental diets

Apple snail eggs were collected from the trunks or stems of the plants or on the walls above the water in rural paddy fields, lotus ponds, and streams in Ya’an city, Sichuan Province, southwest China, in May 2014. The eggs were dried in an oven at lower than 50 °C and then powdered with a grinder to avoid destructing the components in the eggs. The egg powder was sifted and added to the diet at different proportions. The powder content protein, total lipid, total carbohydrate, ash and moisture are 15.05, 1.96, 0.41, 57.26, 5.55 % respectively.

Three experimental treatments were designed as follows: control diet without golden apple snail egg powder; a diet with 5 % egg powder (EP 5 %), and a diet with 15 % egg powder (EP 15 %). The mineral and vitamin premixes used were supplied by Tong Wei Co., Ltd., Chengdu, China. Ingredient contents and proximate composition are shown in Table 1. The basal diet was obtained from Tong Wei Co., Ltd. All dietary ingredients were thoroughly mixed, moistened by adding water, and then minced into pellets. The diets were dried overnight under 25 ± 2 °C and then stored at −20 °C until use.

**Table 1:** Composition of experimental diets fed to Blood parrot powder

	Control	EP5 %	EP15 %
Ingredients (%)			
Fish meal	20	20	20
Soybean meal (43 %)	15.3	15.3	15.3
Rapeseed meal	13	12.5	11.5
Cottonseed meal	10	10	10
Wheat flour	27.7	23.7	15.7
Squid paste	3	3	3
Fish oil	2	2	2
Soybean lecithin oil	2	2	2
Bentonite	3.3	2.8	1.8
Ca (H_2_PO_4_)_2_	2	2	2
Vitamin premix	0.5	0.5	0.5
Mineral premix	1	1	1
Choline chloride	0.2	0.2	0.2
Golden apple snail egg	0	5	15
Chemical composition (%)			
Crude protein	38.1	37.9	37.7
Crude lipid	13.1	13.2	13.2
Ash	14.3	14.3	14.7
Moisture	7.8	7.7	7.7

### Fish rearing condition

Blood parrot with an average weight of 26.53 ± 4.07 g were obtained from a commercial fish farm in Guangdong Province, China. Prior to the experiment, the fish were fed with the basal diet for 2 weeks to acclimatize them to the laboratory culturing system. Ninety Blood parrots were divided into three treatment groups with three replicates per treatment and cultivated in an aquarium with dimensions of 116 cm × 36 cm × 27 cm under continuous aeration. Manual feeding was continued until apparent satiation, twice daily at 9:00 and 17:00. One-third of the water was changed at a specific time daily from 8:00 to 9:00 am, and the temperature was maintained at 27–29 °C. The feeding experiment lasted for 60 days.

### Carotenoid analysis

Nine fish obtained from each treatment group were used for carotenoid analysis. The fish were anesthetized with an dose of MS-222 (about 100 mg/L) before the skin and caudal fin were removed at every 20 days until the end of the experiment. Three other fish were randomly selected for carotenoid analysis as control at the start trial. The removed skin and fin samples were homogenized with a mortar, and 0.05–0.08 g of the samples were obtained for analysis. The samples were then transferred into 10-mL pre-weighed glass tubes and extracted twice. Tissue was weighted and carotenoid extraction done with acetone, until no colour was observed. Partial evaporation of the combined acetone extracts was carried out until approximately 5 mL was attained. Supernate were transfered into another tube. The step was performed again with add approximate 5 mL acetone, afterwards the lower phase (hypophase) was allowed to separate with n-hexane until no colour was observed.

After 24 h, the absorption of the extracts was determined at 480 nm by using a spectrophotometer (Shanghai Metash Instruments CO. LTD, model UV8000A).

Carotenoid content was determined according to the method described by Kalinowski et al. (2007) and calculated using the formula:

$$S = (A \times K \times V)/(E \times G)$$

where *S* is the carotenoid content (mg/kg), *A* is the absorbance, *K* is a constant (10^4^), *V* is the volume of extracting solution (mL), *E* is the extinction coefficient (2500), and *G* is the sample weight (g).

### Chromatophore observation

At the end of the experiment, three to four scales were removed with forceps from the dorsal scale area of each fish and were photographed under a microscope (BX51; Olympus). The number of chromatophores was counted under the microscope at 20× magnification (Van der Salm et al. 2005).

### Antioxidant system assays in liver

The liver of the fishes were homogenized in ice-cold physiological saline to form liver homogenate (1 g tissue in 9 mL physiological saline). The homogenates were then centrifuged at 2500 rpm for 10 min and the supernatants were used for the subsequent tests. The concentration of protein and the activities of SOD and CAT in the live homogenate were assayed using kits which purchased from Nanjing Jiancheng Bioengineering Institute (Nanjing, China).

### Statistical analysis

All values were presented as mean ± standard deviation (SD). Data were analyzed using Two-Way ANOVA to examine the main effects of carotenoid content in same tissue, Fisher’s protected least-significant difference (PLSD) test with the SPSS 17.0 system. Differences were considered significant at the 0.05 level.

## Results

### Survival

After 60 days of the experiment, no mortality occurred throughout the experiment.

### Coloration effect

**Fig. 1:**
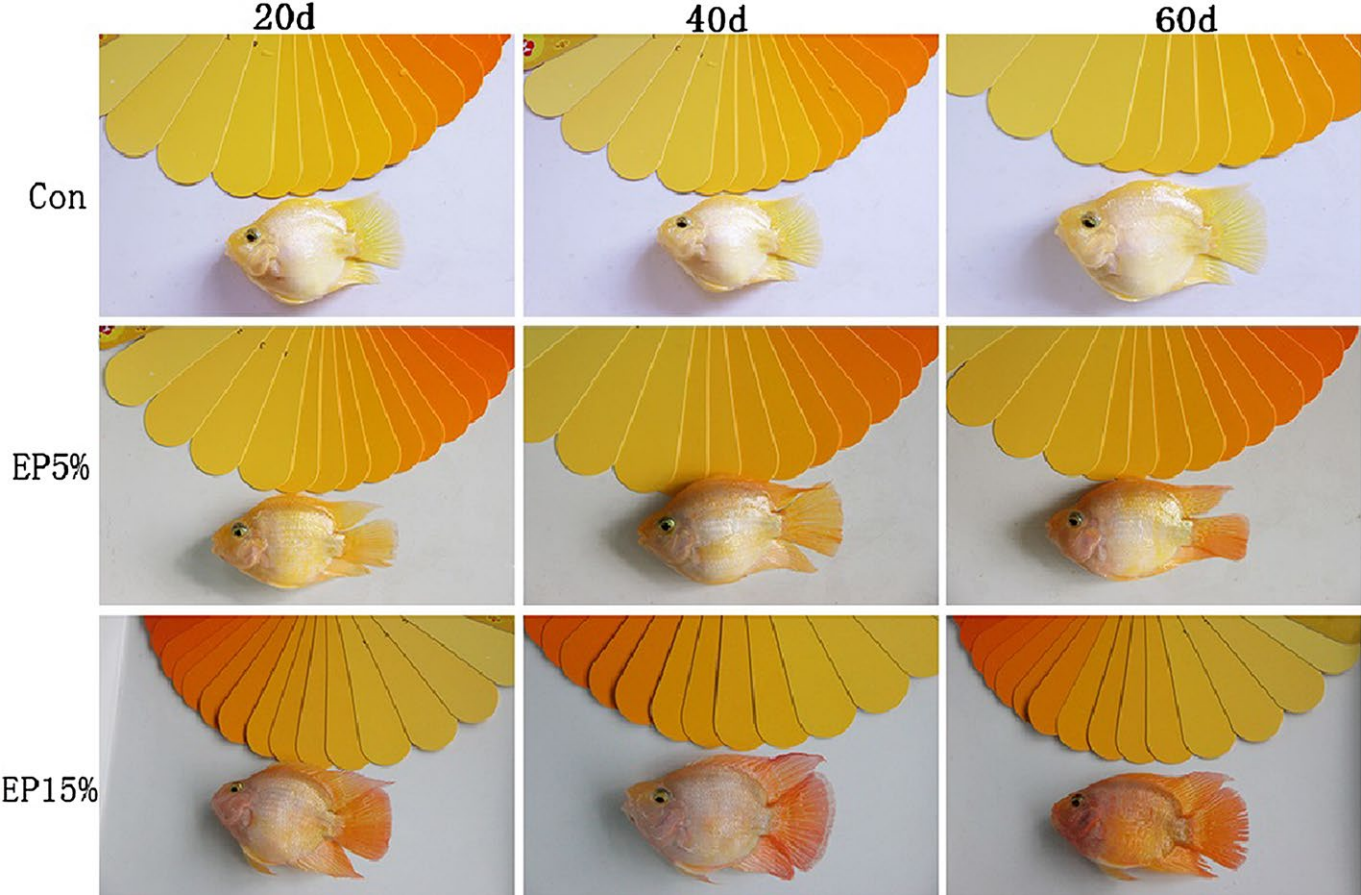
Coloring effects on Blood parrot in each experimental group fed for 20, 40, and 60 days

At the end of diet experiment, an evident coloration effect was observed and is presented in Fig. 1. The body coloration of Blood parrot was enhanced in the skin and caudal fin, particularly in the dorsal fin, caudal fin, anal fin, and both of the body lateral lines, with increasing amount of dry powder of golden apple snail eggs added in the diet.

### Carotenoid concentration

**Fig. 2:**
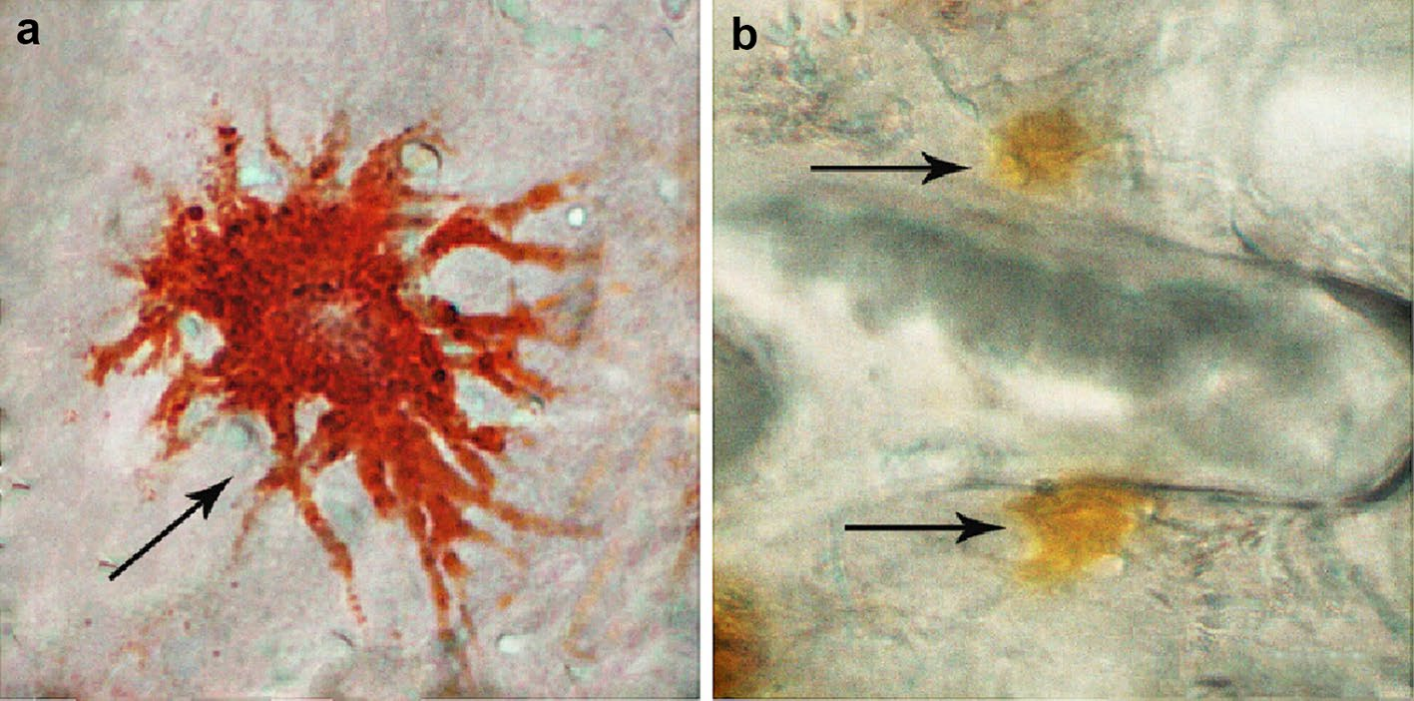
Erythrophores and xanthophores in the scales of blood parrot (40×).** a** Erythrophores (*arrow*).** b** Xanthophores (*arrow*)

After 60 days of the experiment, the carotenoid content was higher in EP (5 %) and EP (15 %) fed with diets supplemented with apple snail egg powder than that in the control group fed without egg powder. The total carotenoid contents in the fish skin and caudal fin are summarized in Table 2. EP (5 %) and EP (15 %) presented increasing trends, in which the carotenoid content in the skin was directly related to the amount of the added egg powder. Conversely, a decreasing trend was observed in the control group (Fig. 2).

**Table 2:** Carotenoid content (mg/kg) in the skin and tail fin of Blood parrot fed the golden apple snail egg powder for 20, 40, and 60 days

	Dietary treatments		
	Control	EP5 %	EP15 %
0 day			
Skin	36.13 ± 10.65^ab^	36.13 ± 10.65^a^	36.13 ± 10.65^a^
Tail fin	50.51 ± 8.33^x^	50.51 ± 8.33^x^	50.51 ± 8.33^x^
20 days			
Skin	52.96 ± 13.46^b^	53.17 ± 16.09^a^	46.59 ± 14.17^ab^
Tail fin	43.45 ± 14.63^x^	62.55 ± 11.75^x^	81.44 ± 17.70*^y^
40 days			
Skin	43.90 ± 14.82^ab^	53.31 ± 9.51^a^	61.38 ± 7.52^b^
Tail fin	35.24 ± 8.78^x^	77.96 ± 18.41*^x^	86.91 ± 30.63*^y^
60 days			
Skin	30.10 ± 8.90^a^	48.94 ± 15.38^a^	60.86 ± 10.83*^b^
Tail fin	21.02 ± 5.86^x^	71.80 ± 29.93*^x^	131.82 ± 23.22*^z^

Values are presented as the mean ± SD

* *P* < 0.05 versus control group, Different superscripts a and b with each columndemarcate significant (*P* < 0.05) differences in the skin tissue; different superscripts x, yandzdemarcate significant (*P* < 0.05) differences in the tail fin tissue

### Chromatophores

**Fig. 3:**
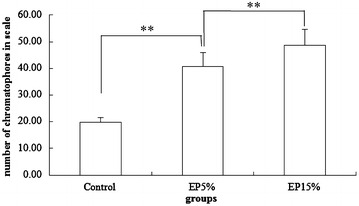
Total number of xanthophores and erythrophores in the scales under the microscope at ×20 (**P < 0.01) after 60 days

Erythrophores and xanthophores were detected in the scale of Blood parrot (Fig. 2). The total number of erythrophores and xanthophores in the scales was evaluated under a microscope at 20×. After 60 days of experiment, the total amount calculated significantly differed (*P* < 0.01) among treatment groups, which was 19.80 ± 1.64 in Control group, 40.75 ± 5.12 in EP 5 % group, and 48.69 ± 5.99 in EP 15 % group (Fig. 3).

### Antioxidant activities

Superoxide dismutase (SOD) and catalase (CAT) activities of the liver of Blood parrot are summarized in Tables 3 and 4. The EP15 % treated fishes showed level significant higher (P < 0.05) in the activities of SOD after 60 days. An increased trend was found (P > 0.05) in CAT activities, a increase trend were founded, especially in SOD activities (Table 3).

**Table 3:** SOD activities (U/mg prot) of the liver of Blood parrot fed the golden apple snail egg for 20, 40 and 60 days

	Dietary treatments		
	Control	EP 5 %	EP 15 %
20 days	51.11 ± 5.31	53.45 ± 20.62	49.77 ± 4.36
40 days	51.76 ± 1.16	55.75 ± 17.39	57.56 ± 9.25
60 days	62.62 ± 3.45	57.79 ± 14.33	88.95 ± 10.96*

Values are presented as the mean ± SD

* *P* < 0.05 versus control group

**Table 4:** CAT activities (U/mg prot) of the liver of Blood parrot fed the golden apple snail egg for 20, 40 and 60 days

	Dietary treatments		
	Control	EP5 %	EP15 %
20 days	75.16 ± 15.25	66.69 ± 18.57	68.40 ± 9.86
40 days	64.44 ± 2.88	54.18 ± 16.89	54.76 ± 20.56
60 days	74.11 ± 12.84	67.28 ± 10.49	101.31 ± 25.52

Values are presented as the mean ± SD

## Discussion

Carotenoids are an important class of pigments in fish, and its concentration in tissues determines the color of the fish (Pham et al. 2014; Sefc et al. 2014). Color is the primary indicator of quality and market price of ornamental fish (Pham et al. 2014). Nonetheless, fish cannot completely synthesize their own carotenoid color, which must be included in their diets (Mills and Patterson 2009; Olson and Owens 1998). Given this limitation, researchers conducted numerous studies on the effects of different carotenoid sources on fish pigmentation. Pham et al. (2014) reported that dietary inclusion of paprika (*Capsicum annum* L.) and *Haematococcus pluvialis* extract with approximately 100 mg/kg carotenoid can increase the total carotenoid skin pigmentation of olive flounder (*Paralichthys olivaceus*). The addition of red pepper (*Capsicum annum* L.) and marigold flower (*Tagetes erecta*) can also increase the pigmentation level of rainbow trout (Büyükçapar et al. 2007). The carotenoid concentration in the skin of red porgy (*Pagrus pagrus*) is augmented with increasing amount of shrimp shell meal diet (Kalinowski et al. 2007). On the other hand, Boonyapakdee et al. (2014) reported that dietary concentrations with 50 mg/kg astaxanthin extracted from golden apple snail eggs can improve the skin pigmentation (L* and b* values) of fancy carp, but in this study, the total carotenoid content in fish was undetermined and whether fish coloration could be improved by feeding with diet EP remains ambiguous.

In the present work, EP was added as a source of natural carotenoid in the diet of Blood parrot. Supplementation of this diet increased the carotenoid concentration in the skin and caudal fin of the fish. The high and low levels of the added EP significantly increased the carotenoid content in the caudal fin of the fish on 20 and 40 days, respectively. Moreover, the carotenoid concentration in the skin was significantly higher in the groups supplemented with high levels of EP for 60 days. The results indicate that EP can improve the pigmentation in the caudal fin, but the effect on the skin is evident at high concentration only. In addition, the carotenoid concentration in the skin and caudal fins of Blood parrot decreased during 20–60 days of supplementing diet without EP, this phenomenon is also observed in other species, such as rainbow trout (Torrissen 1985) and *Amphiprion ocellaris* (Ho et al. 2013). This decreased concentration can be dilution by the growth of the fish (Torrissen 1985).

Fish color is primarily dependent on the presence of chromatophores (melanophores, xanthophores, erythrophores, iridophores, leucophores, and cyanophores) containing pigments, such as melanins, carotenoids (e.g., astaxanthin, canthaxanthin, lutein, and zeaxanthin), pteridines, and purines (Chatzifotis et al. 2011). Carotenoid pigments are stored in xanthophores and erythrophores (yellow and red pigment cells, respectively) (Chatzifotis et al. 2011; Mills and Patterson 2009; Sefc et al. 2014). In the present experiment, the number of xanthophores and erythrophores in the scales was significantly affected by the concentration of apple snail egg powder. This result showed that EP can be efficiently utilized for scale deposition and coloration of Blood parrot. Furthermore, the presence of different types of carotenoids in diet will result in different colors of fish. Yedier reported that astaxanthin in the diet increases the red–orange color in the skin of the red zebra cichlid, whereas Spirulina intake increases the orange and yellow tones (Yedier et al. 2014). In the present study, Blood parrot showed the red–orange color at the end of the experiment. The appearance of this color could be attributed to the main carotenoid content, namely, esterified and non-esterified astaxanthin, in apple snail eggs.

The antioxidant defense system, especial the enzymatic scavengers SOD, CAT and glutathione peroxidase, provides protection against potentially harmful ROS produced constantly during aerobic cell respiration (Lygren et al. 1999). SOD speeds the conversion of superoxide to hydrogen peroxide, whereas CAT and glutathione peroxidase convert hydrogen peroxide to water (Finkel and Holbrook 2000). In some fish, the supplemental dietary vitamin E or carotenoid may have an effect on the antioxidant enzyme activities. Pham et al. (2014) found that SOD activities in liver and plasma of juvenile olive flounder fed diets containing carotenoids were lower than those of the control group. Similar results were observed in juvenile tiger prawn (*Penaeus monodon)* (Chien et al. 2003) and characins (*Hyphessobrycon callistus)* (Wang et al. 2006). Lygren et al. (1999) reported that supplemental diet with high levels of fat-soluble antioxidants (astaxanthin and vitamin E) caused a reduced need for endogenous antioxidant enzymes, such as CAT and total SOD, in protection against H_2_O_2_ and ·O_2_^−^, respectively. However, in this study, SOD and CAT activities of the fish increased after supplementing with 15 % dry powder of golden apple snail egg, which is abundant and rich in carotenoid pigments, for 60 days. The SOD and CAT activities were inconsistent with the reported papers in liver of the fish compared with pure carotenoids.

According to Sun’s reports, more than 59 proteins from the perivitelline fluid (PVF) of *P. canaliculata* had been identified during its embryonic development (Sun et al. 2010, 2012). Among the proteins, ovorubin is a proteinase inhibitor (PI), whose role is to limit predator’s ability to digest egg nutrients. In fact, the antinutritive or antidigestive defense is exactly the egg defensive strategies (Dreon et al. 2010, 2014). Thus, the complex composition of EP may lead to failure of increased antioxidant activity of this fish.

As one of the world’s 100 worst invasive alien species, *P. canaliculata* has gained considerable attention worldwide. Currently, *P. canaliculata* are mainly controlled through physical and biological techniques to benefit the economy, public health, and ecosystems. Utilization of its eggs could destroy the life chains of *P. canaliculata* and thus prevent its spread. Bright-colored golden apple snails crawl out of the water to lay egg masses on plants, concrete walls, and stones above the water surface. These masses are bright pink to red in color and are easy to find and collect. Fresh apple snail eggs can be utilized to extract astaxanthin, but the process is inconvenient as it requires handling of fresh eggs. In the present study, fresh eggs were dried after collection and this method is preferred for storage and utilization of golden apple snail eggs. This technique also exhibits no negative effects on human beings, non-target organisms, and the ecological environment.

## Conclusions

We have found obvious pigmentation effects of supplementing golden apple snail (*Pomacea canaliculata*) eggs powder (EP) in the diet as a source of natural carotenoids on Blood parrot. During a 60-days feeding experiment, no differences in survival of the fish among treatments were found. There were no significant changes in CAT activities (*P* > 0.05), but a significantly higher (*P* < 0.05) in the activities of SOD were observed at the end of experiment in EP 15 % treated fishes after 60 days. The body coloration of Blood parrot was enhanced in the skin and caudal fin with increasing content of golden apple snail egg powder in the diet. At the end of the experiment, the carotenoid content in the caudal fin and the number of scale chromatophores of the fish fed dietary with EP were higher (*P* < 0.05) compared with the control group. Results demonstrated that golden apple snail eggs can be used as a colorant to promote the pigmentation efficacy of Blood parrot.
