# Erratum to: Effects of dietary supplementation of golden apple snail (*Pomacea canaliculata*) egg on survival, pigmentation and antioxidant activity of Blood parrot

**DOI:** 10.1186/s40064-016-3327-6

**Published:** 2016-10-03

**Authors:** Song Yang, Qiao Liu, Yue Wang, Liu-Lan Zhao, Yan Wang, Shi-yong Yang, Zong-jun Du, Jia-en Zhang

**Affiliations:** 1 College of Animal Science and Technology, Sichuan Agricultural University, Chengdu, 611130 Sichuan China; 2 Institute of Tropical and Subtropical Ecology, South China Agricultural University, Guangzhou, 510642 Guangdong China

## Erratum to: SpringerPlus (2016) 5:1556 DOI 10.1186/s40064-016-3051-2

Upon publication of the original article (Yang et al. 2016), it was noticed that the Acknowledgements section was incorrect. The Acknowledgements should actually read:

“This research was funded by the National Nature Science Foundation of China (Grant No. U1131006) and also supported by the “Double support Project” Fund of Sichuan Agricultural University, SICAU (No. 03570202). We are also grateful for the assistance obtained from Jin-wei Yang and Tao Yan of the College of Animal Science and Technology, SICAU, as well as Qiang-qiang Wu of Tongwei Co. Ltd.”
